# Identification of associations between lncRNA and drug resistance based on deep learning and attention mechanism

**DOI:** 10.3389/fmicb.2023.1147778

**Published:** 2023-04-26

**Authors:** Meihong Gao, Xuequn Shang

**Affiliations:** School of Computer Science, Northwestern Polytechnical University, Xi'an, China

**Keywords:** lncRNA-drug resistance associations, deep neural networks, graph attention mechanisms, similarity networks, embeddings

## Abstract

**Introduction:**

Abnormal lncRNA expression can lead to the resistance of tumor cells to anticancer drugs, which is a crucial factor leading to high cancer mortality. Studying the relationship between lncRNA and drug resistance becomes necessary. Recently, deep learning has achieved promising results in predicting biomolecular associations. However, to our knowledge, deep learning-based lncRNA-drug resistance associations prediction has yet to be studied.

**Methods:**

Here, we proposed a new computational model, DeepLDA, which used deep neural networks and graph attention mechanisms to learn lncRNA and drug embeddings for predicting potential relationships between lncRNAs and drug resistance. DeepLDA first constructed similarity networks for lncRNAs and drugs using known association information. Subsequently, deep graph neural networks were utilized to automatically extract features from multiple attributes of lncRNAs and drugs. These features were fed into graph attention networks to learn lncRNA and drug embeddings. Finally, the embeddings were used to predict potential associations between lncRNAs and drug resistance.

**Results:**

Experimental results on the given datasets show that DeepLDA outperforms other machine learning-related prediction methods, and the deep neural network and attention mechanism can improve model performance.

**Dicsussion:**

In summary, this study proposes a powerful deep-learning model that can effectively predict lncRNA-drug resistance associations and facilitate the development of lncRNA-targeted drugs. DeepLDA is available at https://github.com/meihonggao/DeepLDA.

## 1. Introduction

Long non-coding RNA (lncRNA) is a transcript longer than 200 nucleotides, which is transcribed from the genome, cannot be translated into functional proteins, and has heterogeneity in organisms (Mattick and Rinn, 2015; Koch, 2017; Bridges et al., 2021; Zhao et al., 2021). More and more lncRNAs have been proven to affect various biological processes to cause cancer through transcription initiation, transcriptional and post-transcriptional regulation, and are no longer the so-called “transcriptional noise” (Long et al., 2017; Peng et al., 2017; Jiang et al., 2019; Gao et al., 2021, 2022b). In addition, cancer is the leading cause of death worldwide (Bray et al., 2020; Ferlay et al., 2021; Gao and Shang, 2022b; Xia et al., 2022). The current primary therapeutic approach for cancer is chemotherapy, which uses chemical drugs to treat patients. However, tumor cells can become resistant to chemotherapy during treatment, leading to treatment failure (Rebucci and Michiels, 2013; Hu et al., 2019; Rehman et al., 2021). Thus, drug resistance is still a significant challenge in cancer therapy, and its underlying mechanisms have not been fully elucidated.

LncRNAs have recently been identified as a novel mechanism of drug resistance and have received extensive attention in cancer research (Bester et al., 2018; Sun et al., 2019; Barth et al., 2020; Jiang et al., 2020; Singh et al., 2022; Zhou et al., 2022). The abnormal expression of lncRNA can lead to the resistance of tumor cells to anticancer drugs, which is a crucial factor leading to high cancer mortality. For example, lncRNA HOTAIR is found to be upregulated in tumors, such as breast cancer, gastric cancer, esophageal cancer, and leukemia (Xue et al., 2016; Zhu et al., 2022). It not only participates in the formation of multidrug resistance of tumor cells but also is closely related to the degree of malignancy and poor prognosis of tumors. In addition, the overexpression of lncRNA UCA1 in urothelial carcinoma is associated with chemotherapy resistance, and silencing lncRNA UCA1 can inhibit the migration and invasion of non-small cell lung cancer cells and reverse the drug resistance of cancer cells (Wang et al., 2017; Liu et al., 2019). Furthermore, lncRNA DILA1 is associated with the drug resistance of breast cancer cells, which makes cancer cells resistant to tamoxifen by inhibiting the degradation of cyclin D1 (Shi et al., 2020). Overall, there is a close relationship between lncRNA and drug resistance, and the study of lncRNA-drug resistance association becomes crucial.

Some databases have provided experimentally validated lncRNA-drug resistance association data (Dai et al., 2017; Li et al., 2020). However, existing information is small compared with the unknown one. Although biological experiments can identify new lncRNA-drug resistance associations, they are challenging due to high time and financial costs. Computational methods can predict potential associations between lncRNAs and drug resistance, but to our knowledge, only two related works have been proposed. One is LRGCPND (Li et al., 2021), which infers the relationship between noncoding RNAs and drug resistance based on linear residual graph convolution. The other is GSLRDA (Zheng J. et al., 2022), which uses light graph convolutional networks (GCNs), data augmentation, and self-supervision to identify associations between ncRNAs and drug resistance. There is still much for exploration in lncRNA-drug resistance association prediction. In recent years, machine learning has achieved remarkable results in predicting biomolecular association, such as lncRNA-gene association (Zhang et al., 2020; Zhao et al., 2020; Gao and Shang, 2022a; Gao et al., 2022a), lncRNA-miRNA association (Liu et al., 2020; Zhang et al., 2021a,b), miRNA-drug resistance association (Huang et al., 2020; Niu et al., 2022; Zheng K. et al., 2022), and ncRNA-drug resistance association (Li et al., 2021; Zheng J. et al., 2022). Inspired by this, machine learning methods can be used to predict potential lncRNA-drug resistance associations to explore the impact of lncRNAs on the drug resistance of cancer cells.

In this study, we proposed a deep learning-based computational model, DeepLDA, which used deep neural network and graph attention mechanism to learn embeddings of lncRNAs and drugs for predicting potential lncRNA-drug resistance associations. We first used the known relationship between lncRNAs and drug resistance to construct similarity networks for lncRNAs and drugs. Subsequently, deep GCN were used to automatically extract features from multiple attributes of the raw data of nodes. These features were then used as input to graph attention network (GAT) module for embedding learning. Finally, lncRNA and drug embeddings were used to predict potential associations between lncRNAs and drug resistance. Experimental results show that the prediction performance of DeepLDA is better than other ncRNA-drug resistance association prediction methods, and the deep neural network and attention mechanism are proved to improve the model performance. In summary, this study proposes a new computational model that can effectively complete the task of lncRNA-drug resistance association prediction, help to understand the lncRNA-related drug resistance mechanism, accelerate drug development, and promote the development of targeted therapy.

## 2. Materials and methods

**Figure 1:**
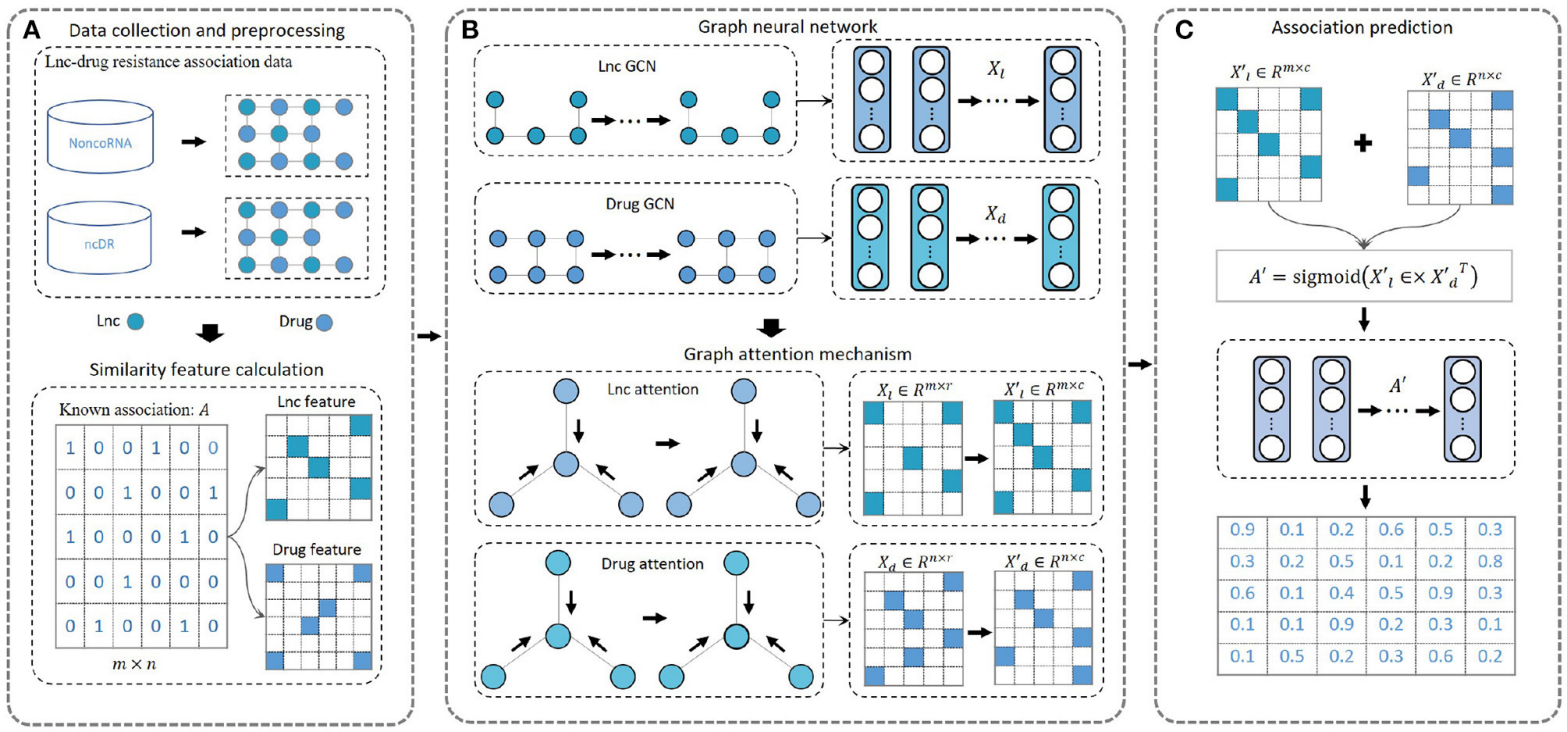
Overview of DeepLDA. **(A)** Known lncRNA-drug resistance association network. **(B)** Graph neural networks are used to initially learn lncRNA and drug features. Graph attention mechanism are used to learn lncRNA and drug embeddings. **(C)** Using learned embeddings to predict association scores for lncRNA-drug resistance items.

We designed a new computational model, DeepLDA, to predict candidate lncRNA-drug associations based on deep learning and graph attention mechanism (Figure 1). Firstly, lncRNA-drug resistance association data were collected and preprocessed (Figure 1A). Then, we proposed a deep learning module based on graph neural network and graph attention mechanism to learn lncRNA and drug embeddings (Figure 1B). Finally, these learned embeddings were used to identify potential associations between lncRNAs and drug resistance (Figure 1C).

### 2.1. Data collection and experimental setup

LncRNAs-drug resistance associations were collected from NoncoRNA (Li et al., 2020) and ncDR (Dai et al., 2017) datasets. Here, experimentally validated association terms were extracted for subsequent prediction analysis. After preprocessing, these items are transformed into association networks (Table 1). As we can see in Table 1, the number of lncRNAs, drugs, and lncRNA-drug resistance associations in the NoncoRNA-related association network are 3,601, 71, and 3,802, respectively. In the ncDR-related association network, the numbers of lncRNAs, drugs and lncRNA-drug resistance associations are 162, 31, and 184, respectively.

**Table 1:** Details of lncRNA-drug resistance associations.

**Dataset**	**LncRNA**	**Drug**	**LncRNA-drug resistance**
NoncoRNA	3,599	71	3,802
ncDR	162	31	184

The model was evaluated on balanced and unbalanced datasets. On the unbalanced dataset, we used all LncRNA-drug association items for a more practical simulation. Here, all negative samples were selected for training and testing. Due to the limited understanding of the regulatory relationship between lncRNAs and drug resistances, the number of positive samples was much lower than that of negative samples, which resulted in an imbalance of samples. On balanced datasets, the same amount of negative samples was sampled as the positive sample before training and testing. During training and testing, we performed 10-fold cross-validation, as shown in our previous study (Gao et al., 2022a). Finally, four metrics, the AUC, AUPR, F1-score, and MCC, were calculated to evaluate model performance.

### 2.2. Similarity calculation

We obtained lncRNA and drug features through lncRNA-drug resistance association information. Specifically, we assumed that lncRNAs with similar functions have similar drug resistance patterns. The similarity between lncRNAs was calculated using the Gaussian kernel function as follows:


(1) $$\begin{array}{l} G_{l}\left(i,j\right)=\exp\left(-\alpha_{l}∥A\left(i,:\right)-A\left(j,:\right){∥}^{2}\right) \end{array}$$

(2) $$\begin{array}{l} \alpha_{l}=\frac{1}{m}\overset{m}{\underset{k=1}{\sum }}∥A\left(k,:\right){∥}^{2} \end{array}$$

where *A* ∈ *R*^*m*×*n*^ represents the known lncRNA-drug resistance associations, ∥*X*∥ represents the Euclidean distance from *X* to the origin, and *m* and *n* represent the number of lncRNAs and drugs related to the association network, respectively. Similarly, we calculated the similarity between drugs as follows:


(3) $$\begin{array}{l} G_{d}\left(i,j\right)=\exp\left(-\alpha_{d}∥A\left(:,i\right)-A\left(:,j\right){∥}^{2}\right) \end{array}$$

(4) $$\begin{array}{l} \alpha_{d}=\frac{1}{n}\overset{n}{\underset{k=1}{\sum }}∥A\left(:,k\right){∥}^{2} \end{array}$$

Finally, we obtained similarity features $G_{l}\in R^{m\times m}$ for lncRNAs and $G_{d}\in R^{n\times n}$ for drugs.

### 2.3. Embedding learning

We designed a deep learning module based on graph neural network and graph attention mechanism to learn lncRNA and drug embeddings (Figure 1B). GCN was used to initially extract lncRNA features, and its layer propagation formula is as follows:


(5) $$\begin{array}{l} X_{l}=\text{softmax}\left(\hat{A_{l}}\text{ReLU}\left(\hat{A_{l}}XW_{l}^{\left(0\right)}\right)W_{l}^{\left(1\right)}\right) \end{array}$$

where $\hat{A_{l}}={\tilde{D_{l}}}^{-\frac{1}{2}}\tilde{A_{l}}{\tilde{D_{l}}}^{-\frac{1}{2}}$, Ã_*l*_ = *G*_*l*_ + *I* and I represents the identity matrix, ${\tilde{D}}_{l}$ represents the degree matrix of matrix Ã_*l*_, and $W_{l}^{\left(0\right)}$ and $W_{l}^{\left(1\right)}$ represents layer-specific weight matrices. Further, we learned lncRNA embeddings via the graph attention mechanism. Here, the input to the GAT is $X_{l}\in R^{m\times r}=\left\{{\vec{x}}_{l_{1}},{\vec{x}}_{l_{2}},\cdots \;,{\vec{x}}_{l_{m}}\right\}$, and the attention coefficient between lncRNA *l*_*i*_ and lncRNA *l*_*j*_ is defined as follows:


(6) $$\begin{array}{l} e_{l_{i}l_{j}}=a\left(W_{l}{\vec{x}}_{l_{i}},W_{l}{\vec{x}}_{l_{j}}\right) \end{array}$$

where $W_{l}\in R^{r\times c}$ is a parameter matrix, and *a* is a projection: *R*^*c*×*c*^ → *R*. Furthermore, we normalized *e*_*l*_*i*_*l*_*j*__ across all choices of *l*_*j*_ as follows:


(7) $$\begin{array}{l} \alpha_{l_{i}l_{j}}=\text{softma}{\text{x}}_{l_{j}}\left(e_{l_{i}l_{j}}\right)=\frac{\text{exp}\left(e_{l_{i}l_{j}}\right)}{\sum_{l_{k}\in N_{l_{i}}}\text{exp}\left(e_{l_{i}l_{k}}\right)} \end{array}$$

where *N*_*l*_*i*__ is the neighbor of node *l*_*i*_ and α_*l*_*i*_*l*_*j*__ can be fully expanded as:


(8) $$\begin{array}{l} \alpha_{l_{i}l_{j}}=\frac{\text{exp}\left(\text{LeakyReLU}\left({\vec{a}}^{T}\left[W_{l}{\vec{x}}_{l_{i}}||W_{l}{\vec{x}}_{l_{j}}\right]\right)\right)}{\sum_{l_{k}\in N_{l_{i}}}\text{exp}\left(\text{LeakyReLU}\left({\vec{a}}^{T}\left[W_{l}{\vec{x}}_{l_{i}}∥W_{l}{\vec{x}}_{l_{k}}\right]\right)\right)} \end{array}$$

where $\vec{a}\in R^{2c}$ is a weight vector and || is the concatenation operation. Based on this, we obtained the output feature of *l*_*i*_ as:


(9) $$\begin{array}{l} {\vec{x}}_{l_{i}}^{\prime }=\sigma \left(\underset{l_{j}\in N\left(l_{i}\right)}{\sum }\alpha_{l_{i}l_{j}}W_{l}{\vec{x}}_{l_{j}}\right) \end{array}$$

where ${\vec{x}}_{l_{i}}^{\prime }$ was further calculated by the multi-head attention mechanism as:


(10) $$\begin{array}{l} {\vec{x}}_{l_{i}}^{\prime }=\overset{K}{\underset{k=1}{\left|\right|}}\sigma \left(\underset{l_{j}\in N\left(l_{i}\right)}{\sum }\alpha_{l_{i}l_{j}}^{k}W_{l}^{k}{\vec{x}}_{l_{j}}\right) \end{array}$$

where *K* is the number of heads, $\alpha_{l_{i}l_{j}}^{k}$ is computed by the *k*-th head, and $W_{l}^{k}$ is the corresponding weight matrix. Specially, the multi-head attention mechanism in the last layer is:


(11) $$\begin{array}{l} {\vec{x}}_{l_{i}}^{\prime }=\sigma \left(\frac{1}{K}\overset{K}{\underset{k=1}{\sum }}\underset{l_{j}\in N\left(l_{i}\right)}{\sum }\alpha_{l_{i}l_{j}}^{k}W^{k}{\vec{x}}_{l_{j}}\right) \end{array}$$

After the above operation, lncRNA embeddings are expressed as $X_{l}^{\prime }\in R^{m\times c}=\left\{{\vec{x}}_{l_{1}}^{\prime },{\vec{x}}_{l_{2}}^{\prime },\cdots \;,{\vec{x}}_{l_{m}}^{\prime }\right\}$.

Similar to the lncRNA embeddings learning process, drug features were initially extracted by GCN, which layer propagation formula is:


(12) $$\begin{array}{l} X_{d}=\text{softmax}\left(\hat{A_{d}}\text{ReLU}\left(\hat{A_{d}}X^{\prime }W_{d}^{\left(0\right)}\right)W_{d}^{\left(1\right)}\right) \end{array}$$

where $\hat{A_{d}}={\tilde{D_{d}}}^{-\frac{1}{2}}\tilde{A_{d}}{\tilde{D_{d}}}^{-\frac{1}{2}}$, Ã_*d*_ = *G*_*d*_ + *I*, ${\tilde{D}}_{d}$ represents the degree matrix of matrix Ã_*d*_, and $W_{d}^{\left(0\right)}$ and $W_{d}^{\left(1\right)}$ represents layer-specific weight matrices. Through the above operations, we obtained drug features $X_{d}\in R^{n\times r}$. Further, we learned drug embeddings through graph attention mechanism which input is $X_{d}=\left\{{\vec{x}}_{d_{1}},{\vec{x}}_{d_{2}},\cdots \;,{\vec{x}}_{d_{m}}\right\}$. Then the attention coefficien between drug *d*_*i*_ and drug *d*_*j*_ is:


(13) $$\begin{array}{l} e_{d_{i}d_{j}}=a\left(W_{d}{\vec{x}}_{d_{i}},W_{d}{\vec{x}}_{d_{j}}\right) \end{array}$$

where $W_{d}\in R^{r\times c}$ is a parameter matrix. Furthermore, we normalized *e*_*d*_*i*_*d*_*j*__ across all choices of *d*_*j*_ as follows:


(14) $$\begin{array}{l} \alpha_{d_{i}d_{j}}=\text{softma}{\text{x}}_{d_{j}}\left(e_{d_{i}d_{j}}\right)=\frac{\text{exp}\left(e_{d_{i}d_{j}}\right)}{\sum_{d_{k}\in N_{d_{i}}}\text{exp}\left(e_{d_{i}d_{k}}\right)} \end{array}$$

where *N*_*d*_*i*__ is the neighbor of node *d*_*i*_. The above formula is fully expanded as follows:


(15) $$\begin{array}{l} \alpha_{d_{i}d_{j}}=\frac{\text{exp}\left(\text{LeakyReLU}\left({\vec{a_{d}}}^{T}\left[W_{d}{\vec{x}}_{d_{i}}||W_{d}{\vec{x}}_{d_{j}}\right]\right)\right)}{\sum_{d_{k}\in N_{d_{i}}}\text{exp}\left(\text{LeakyReLU}\left({\vec{a_{d}}}^{T}\left[W_{d}{\vec{x}}_{d_{i}}∥W_{d}{\vec{x}}_{d_{k}}\right]\right)\right)} \end{array}$$

where $\vec{a_{d}}\in R^{2c}$ is a weight vector. Based on this, we obtained the output feature of *d*_*i*_ as follows:


(16) $$\begin{array}{l} {\vec{x}}_{d_{i}}^{\prime }=\sigma \left(\underset{d_{j}\in N\left(d_{i}\right)}{\sum }\alpha_{d_{i}d_{j}}W_{d}{\vec{x}}_{d_{j}}\right) \end{array}$$

where ${\vec{x}}_{d_{i}}^{\prime }$ was calculated by the multi-head attention mechanism as follows:


(17) $$\begin{array}{l} {\vec{x}}_{d_{i}}^{\prime }=\overset{K}{\underset{k=1}{\left|\right|}}\sigma \left(\underset{d_{j}\in N\left(d_{i}\right)}{\sum }\alpha_{d_{i}d_{j}}^{k}W_{d}^{k}{\vec{x}}_{d_{j}}\right) \end{array}$$

where $\alpha_{d_{i}d_{j}}^{k}$ is computed by the *k*-th head and $W_{d}^{k}$ is the corresponding weight matrix. Specially, the multi-head attention mechanism in the last layer is as follows:


(18) $$\begin{array}{l} {\vec{x}}_{d_{i}}^{\prime }=\sigma \left(\frac{1}{K}\overset{K}{\underset{k=1}{\sum }}\underset{d_{j}\in N\left(d_{i}\right)}{\sum }\alpha_{d_{i}d_{j}}^{k}W^{k}{\vec{x}}_{d_{j}}\right) \end{array}$$

Finally, we obtained lncRNA embeddings as $X_{d}^{\prime }\in R^{n\times c}=\left\{{\vec{x}}_{d_{1}}^{\prime },{\vec{x}}_{d_{2}}^{\prime },\cdots \;,{\vec{x}}_{d_{n}}^{\prime }\right\}$.

### 2.4. Association prediction

After obtaining lncRNA and drug embeddings, we used them to predict potential lncRNA-drug resistance associations. The association score is equal to $A^{\prime }=\sigma \left(X_{l}^{\prime }\times {X_{d}^{\prime }}^{\text{T}}\right)$ and σ is a sigmoid function. To make the prediction result as close as possible to the real relationship between lncRNA and drug resistance, the reconstruction loss is defined as follows:


(19) $$\begin{array}{l} Loss=\overset{m}{\underset{i=1}{\sum }}\overset{n}{\underset{j=1}{\sum }}\left(A_{ij}^{\prime }-A_{ij}\right) \end{array}$$

## 3. Results

### 3.1. Parameter analysis

**Figure 2:**
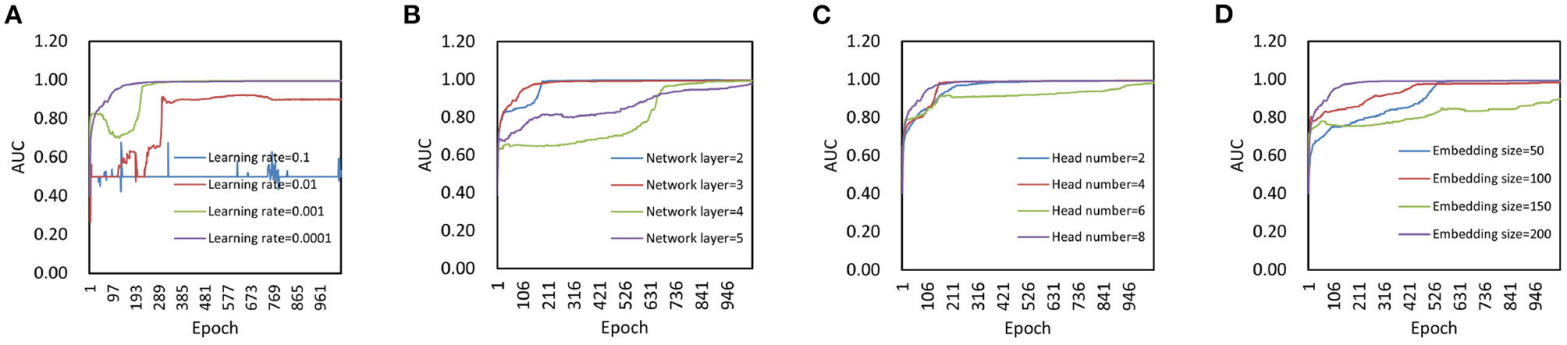
Parameter analysis. **(A)** Effect of learning rate on model performance. **(B)** Effect of network layer on model performance. **(C)** Effect of head number on model performance. **(D)** Effect of embedding size on model performance.

We set the parameters learning rate, network layer, head number, and embedding size in the model as follows. We first changed the learning rate in {0.1, 0.01, 0.001, 0.0001} to determine its effect on model performance (Figure 2A). As we can found, when the learning rate is equal to 0.1, the model is difficult to converge. When the learning rate is in {0.01, 0.001}, the model does not achieve optimal performance. Therefore, 0.0001 is used as the learning rate value. Then, we changed the network layer in {2, 3, 4, 5} to determine its effect on model performanc (Figure 2B). It can be found that a small number of layers can speed up the convergence of the model, and a large number of layers will make the model prone to overfitting. In this experiment, we choose 3 as the network layer.

After that, we compared the model performance with different head number in graph attention mechanism module to determine its impact on model performance. Specifically, we changed the head number in {2, 4, 6, 8} for analysis (Figure 2C). As we can see, there is not much difference in final performance between models with different head number. A large number of attention head can speed up the convergence of the model, and a small number of attention head will make the model converge slowly. In this experiment, we make the head number of the attention mechanism equal to 8. Finally, we set the embedding size in {10, 50, 100, 200} to verify its impact on model prediction performance (Figure 2D). When the embedding size is set to 200, the final AUC is slightly larger than that of the other groups. Thus, we choose 200 as the embedding size.

### 3.2. Performance evaluation

**Figure 3:**
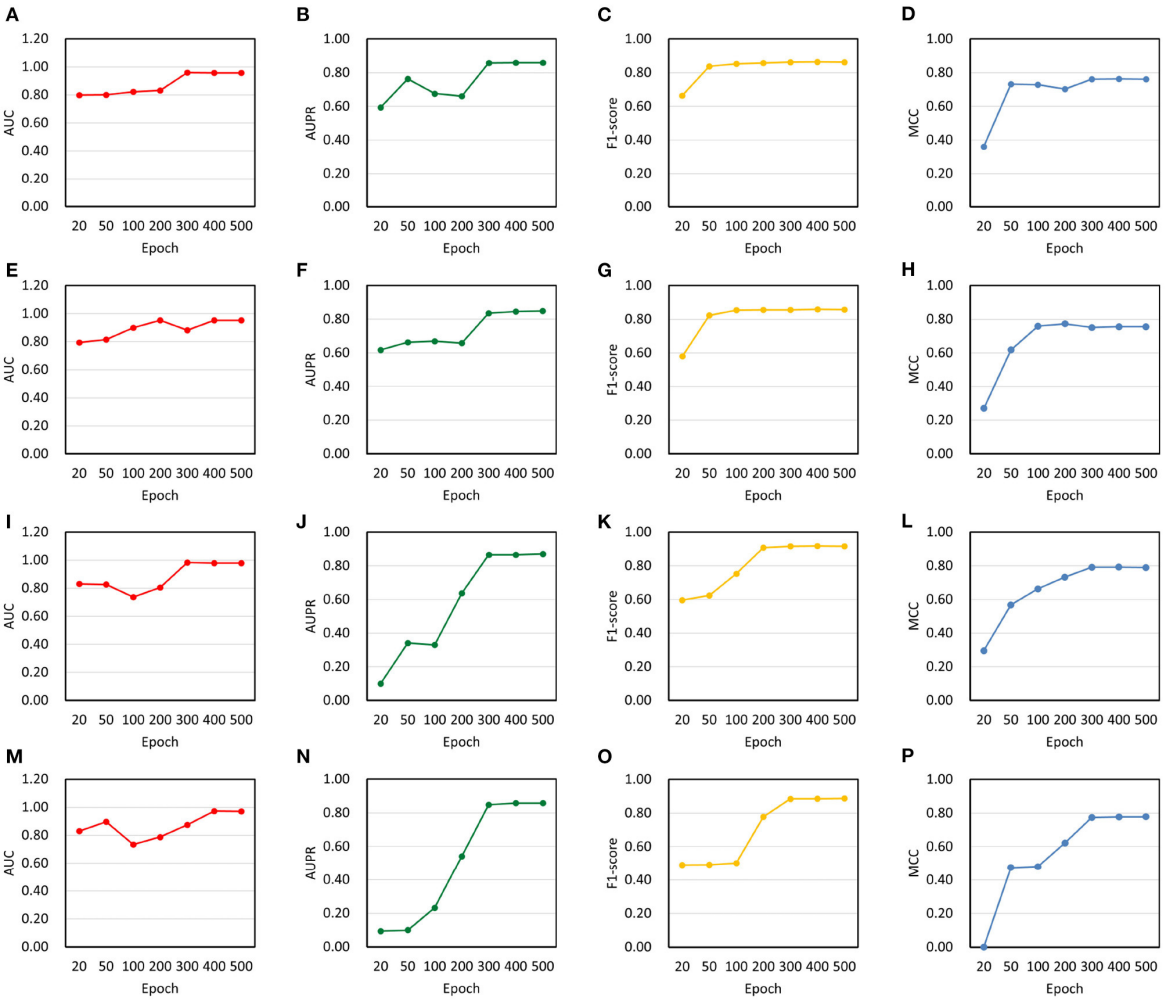
Changes in model performance on balanced and unbalanced datasets. **(A)** AUC on balanced NoncoRNA. **(B)** AUPR on balanced NoncoRNA. **(C)** F1-score on balanced NoncoRNA. **(D)** MCC on balanced NoncoRNA. **(E)** AUC on unbalanced NoncoRNA. **(F)** AUPR on unbalanced NoncoRNA. **(G)** F1-score on unbalanced NoncoRNA. **(H)** MCC on unbalanced NoncoRNA. **(I)** AUC on balanced ncDR. **(J)** AUPR on balanced ncDR. **(K)** F1-score on balanced ncDR. **(L)** MCC on balanced ncDR. **(M)** AUC on unbalanced ncDR. **(N)** AUPR on unbalanced ncDR. **(O)** F1-score on unbalanced ncDR. **(P)** MCC on unbalanced ncDR.

In order to evaluate model performance, we analyzed changes in AUC, AUPR, F1-score and MCC on NoncoRNA and ncDR. As a result, four experimental groups were obtained, including balanced NoncoRNA, balanced ncDR, unbalanced NoncoRNA, and unbalanced ncDR (Figure 3). It can be found that the AUC, AUPR, F1-score and MCC of DeepLDA are stable at 0.96, 0.86, 0.86, and 0.76, respectively, on balanced NoncoRNA (Figures 3A–D). On unbalanced NoncoRNA, the AUC, AUPR, F1 score and MCC of DeepLDA are stable at 0.95, 0.85, 0.86, and 0.76, respectively (Figures 3E–H). In addition, the AUC, AUPR, F1-score and MCC of DeepLDA are stable at 0.98, 0.87, 0.92, and 0.79, respectively, on balanced ncDR (Figures 3I–L). On unbalanced ncDR, the AUC, AUPR, F1 score and MCC of DeepLDA are stable at 0.97, 0.86, 0.89, and 0.78, respectively (Figures 3M–P). The results show that the model performance on the balanced dataset is slightly better than that on the unbalanced dataset. The phenomenon is caused by the proportion of samples in the dataset. Since the number of positive and negative samples in the balanced datasets is the same, the bias caused by unbalanced samples can be eliminated to a certain extent in the prediction task. Thus, our method performs better on balanced datasets than on unbalanced datasets. For unbalanced datasets, although the prediction performance is slightly inferior to the balanced datasets, it is still at a high level, which proves the robustness of our model.

We further calculated average performance metrics, which is equal to the average value of AUC, AUPR, F1-score and MCC (Table 2). It can be found that when the epoch is around 300, the average metrics are close to convergence on the balanced dataset, and when the epoch is around 400, the average metrics are close to convergence on the unbalanced dataset. Specifically, the average metrics on NoncoRNA- and ncDR-related balanced datasets are stable at 0.86 and 0.89, respectively, and the average metrics on NoncoRNA- and ncDR-related unbalanced datasets are stable at 0.85 and 0.87, respectively. The above experimental results demonstrate that our model has satisfactory performance in predicting lncRNA-drug resistance associations and converges faster on balanced datasets than on unbalanced datasets since the data size of balanced datasets is smaller than that of unbalanced datasets. Moreover, the results further verify that the model performance better on the balanced dataset than on the unbalanced dataset, and that the model can also achieve ideal performance on unbalanced datasets.

**Table 2:** Changes in average model performance on balanced and unbalanced datasets.

**Epoch**	**NoncoRNA-balanced**	**NoncoRNA-unbalanced**	**ncDR-balanced**	**ncDR-unbalanced**
20	0.6042	0.5660	0.4553	0.3535
50	0.7835	0.7301	0.5897	0.4908
100	0.7696	0.7959	0.6204	0.4872
200	0.7634	0.8099	0.7707	0.6825
300	0.8608	0.8315	0.8889	0.8454
400	0.8617	0.8534	0.8890	0.8736
500	0.8605	0.8538	0.8890	0.8734

### 3.3. Effect of each module

**Figure 4:**
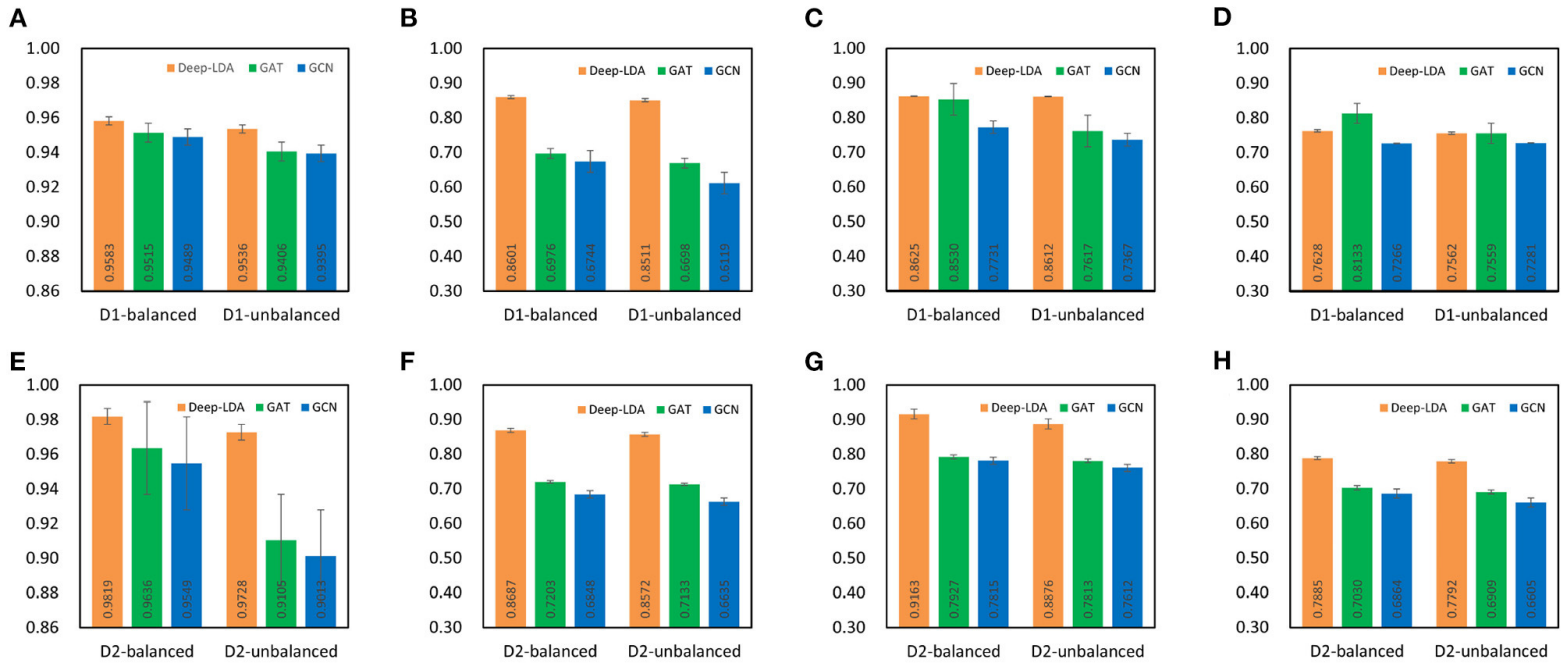
Comparison of the effects of each module. D1 and D2 represent NoncoRNA and ncDR, respectively. **(A)** AUC on D1. **(B)** AUPR on D1. **(C)** F1-score on D1. **(D)** MCC on D1. **(E)** AUC on D2. **(F)** AUPR on D2. **(G)** F1-score on D2. **(H)** MCC on D2.

To demonstrate the effectiveness of GCN and GAT on the lncRNA-drug resistance association prediction task, we compared the performance of GCN, GAT, and DeepLDA under the same experimental setting. As a result, we find that DeepLDA outperforms GAT and GCN on the given datasets (Figure 4). The AUC, AUPR, F1-score, and MCC of DeepLDA are 0.9583, 0.8601, 0.8625, and 0.7628, respectively, on balanced NoncoRNA (Figures 4A–D), are 0.9536, 0.8511, 0.8612, and 0.7562, respectively, on unbalanced NoncoRNA (Figures 4A–D), are 0.9819, 0.8687, 0.9163, and 0.7885, respectively, on balanced ncDR (Figures 4E–H), and are 0.9728, 0.8572, 0.8876, and 0.7792, respectively, on unbalanced ncDR (Figures 4E–H). Through comparative analysis, it can be found that the AUCs of DeepLDA on balanced NoncoRNA, unbalanced NoncoRNA, balanced ncDR, and unbalanced ncDR are 0.0071, 0.0138, 0.0190, and 0.0684 higher than the best AUC in GAT and GCN, respectively. The AUPRs of DeepLDA are 0.2329, 0.2707, 0.2060, and 0.2017 higher than the best AUPR in GAT and GCN on balanced NoncoRNA, unbalanced NoncoRNA, balanced ncDR, and unbalanced ncDR, respectively. The F1-scores of DeepLDA are 0.0111, 0.1306, 0.1559, 0.1361 higher than the best F1-score in GAT and GCN on balanced NoncoRNA, unbalanced NoncoRNA, balanced ncDR, and unbalanced ncDR, respectively. The MCCs of DeepLDA is 0.0004, 0.1216, and 0.1278, higher than the best MCC in GAT and GCN on unbalanced NoncoRNA, balanced ncDR, and unbalanced ncDR, respectively. At the same time, the MCC of DeepLDA is higher than GCN and slightly lower than GAT on balanced NoncoRNA. As for the average performance, which are 0.8609, 0.8555, 0.8889, and 0.8742 on balanced NoncoRNA, unbalanced NoncoRNA, balanced ncDR, and unbalanced ncDR, respectively (Table 3). Compared with the best average performance in GAT and GCN, the improvements are 0.0387, 0.0940, 0.1182, and 0.1295, respectively.

**Table 3:** Comparison of average performance metrics of each module.

**Method**	**Balanced noncoRNA**	**Unbalanced noncoRNA**	**Balanced ncDR**	**Unbalanced ncDR**
Deep-LDA	0.8609	0.8555	0.8889	0.8742
GAT	0.8289	0.7820	0.7949	0.7740
GCN	0.7808	0.7541	0.7769	0.7466

Overall, DeepLDA has an advantage over GAT and GCN in predicting lncRNA-drug resistance associations because DeepLDA combines the feature learning capabilities of GCN and GAT to capture the local and global features of nodes effectively. In addition, GAT has a strong learning ability and generalization ability, and can handle complex and variable lncRNA-drug resistance association data only by learning association-related nodes and their neighbor information, which significantly improves the model prediction performance.

### 3.4. Comparison with other methods

**Figure 5:**
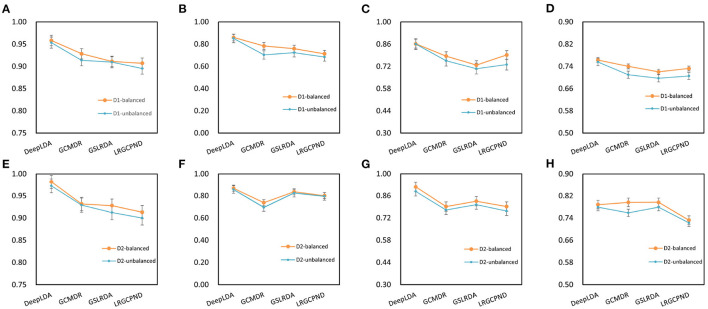
Performance comparison with other methods. D1 and D2 represent NoncoRNA and ncDR, respectively. **(A)** AUC on D1. **(B)** AUPR on D1. **(C)** F1-score on D1. **(D)** MCC on D1. **(E)** AUC on D2. **(F)** AUPR on D2. **(G)** F1-score on D2. **(H)** MCC on D2.

**Figure 6:**
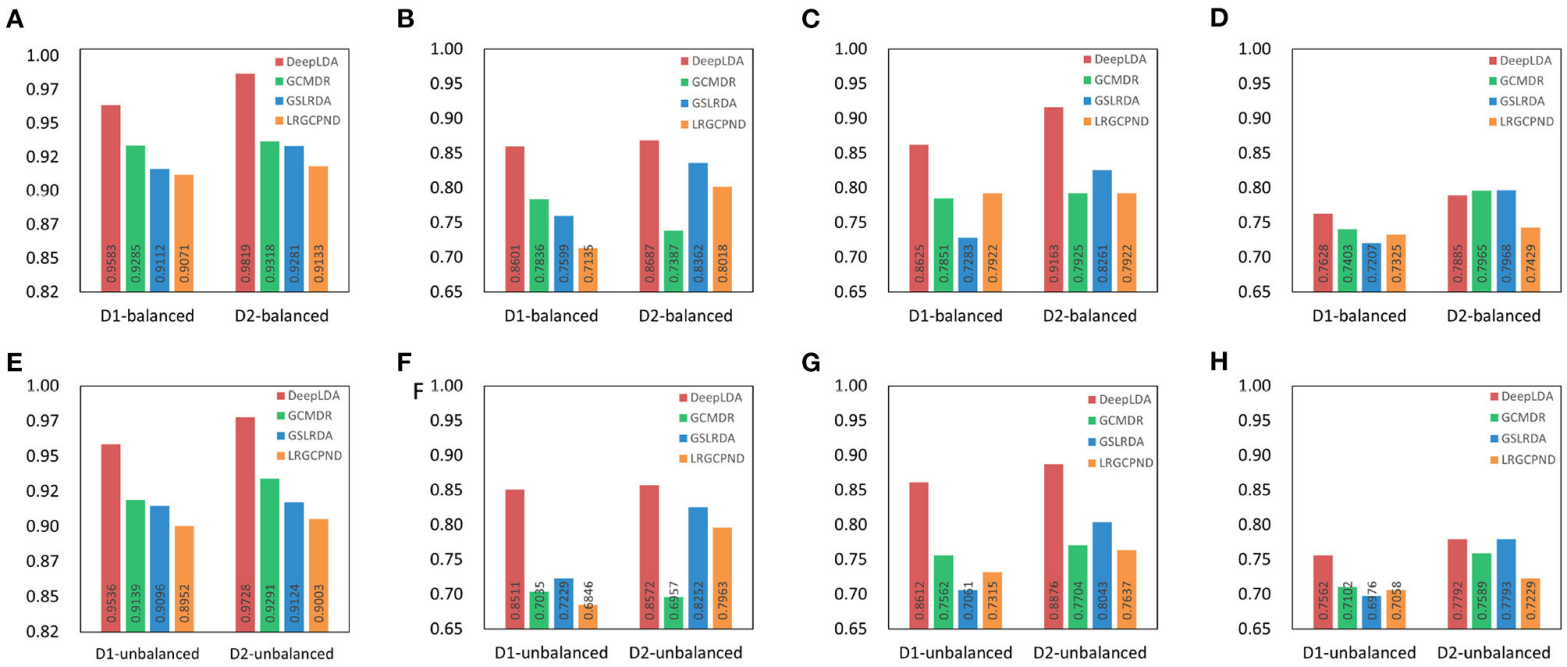
Performance comparison with other methods on balanced and unbalanced datasets. D1 and D2 represent NoncoRNA and ncDR, respectively. **(A)** AUC on balanced datasets. **(B)** AUPR on balanced datasets. **(C)** F1-score on balanced datasets. **(D)** MCC on balanced datasets. **(E)** AUC on unbalanced datasets. **(F)** AUPR on unbalanced datasets. **(G)** F1-score on unbalanced datasets. **(H)** MCC on unbalanced datasets.

We compared the performance of DeepLDA with other association prediction methods, GSLRDA (Zheng J. et al., 2022), LRGCPND (Li et al., 2021), and GCMDR (Huang et al., 2020), to verify its effectiveness. Among them, GSLRDA and LRGCPND were designed to predict ncRNA-drug resistance associations, and GCMDR was designed to predict miRNA-drug resistance associations. As a result, these methods perform better in predicting lncRNA-drug resistance associations on balanced datasets than on unbalanced datasets, as we expected (Figure 5). For dataset NoncoRNA and ncDR, DeepLDA performs better on ncDR than NoncoRNA (Figure 6). As we can see in Figures 5, 6, DeepLDA outperforms other prediction methods in AUC, AUPR, F1-score, and MCC. The AUC of DeepLDA is 0.0321, 0.0538, 0.0434, and 0.0470, better than the second-best method on balanced NoncoRNA, balanced ncDR, unbalanced NoncoRNA and unbalanced ncDR, respectively. The AUPR of DeepLDA is 0.0976, 0.0389, 0.1773, and 0.0388, better than the second-best method on balanced NoncoRNA, balanced ncDR, unbalanced NoncoRNA, and unbalanced ncDR, respectively. The F1-score of DeepLDA is 0.0887, 0.1092, 0.1389, and 0.1036, better than the second-best method on balanced NoncoRNA, balanced ncDR, unbalanced NoncoRNA and unbalanced ncDR, respectively. The MCC of DeepLDA is 0.0.0304 and 0.0648 better than the second-best method on balanced NoncoRNA and unbalanced NoncoRNA, respectively. At the same time, the MCC of DeepLDA is slightly inferior to the second-best method on balanced ncDR and unbalanced ncDR, respectively.

To further demonstrate the effectiveness of DeepLDA, we compared the average performance of different prediction methods (Table 4). As we can see in Table 4, the average performance of GCMDR on balanced NoncoRNA, unbalanced NoncoRNA, balanced ncDR, and unbalanced ncDR are 0.8094, 0.7710, 0.8149, and 0.7885 respectively. The average performance of GSLRDA on balanced NoncoRNA, unbalanced NoncoRNA, balanced ncDR, and unbalanced ncDR are 0.7800, 0.7591, 0.8468, and 0.8303, respectively. As for LRGCPND, its average performance on balanced NoncoRNA, unbalanced NoncoRNA, balanced ncDR, and unbalanced ncDR are 0.7863, 0.7543, 0.8126, and 0.7958, respectively. Comparative analysis shows that the average performance of DeepLDA on balanced NoncoRNA, unbalanced NoncoRNA, balanced ncDR, and unbalanced ncDR are 0.0637, 0.1097, 0.0497, and 0.0529 better than the suboptimal method, respectively.

**Table 4:** Comparison of average performance metrics of different methods.

**Method**	**Balanced noncoRNA**	**Unbalanced noncoRNA**	**Balanced ncDR**	**Unbalanced ncDR**
Deep-LDA	0.8609	0.8555	0.8889	0.8742
GCMDR	0.8094	0.7710	0.8149	0.7885
GSLRDA	0.7800	0.7591	0.8468	0.8303
LRGCPND	0.7863	0.7543	0.8126	0.7958

Overall, the performance of DeepLDA is significantly superior to GSLRDA, LRGCPND, and GCMDR. The performance advantage of DeepLDA is attributed to the following two points. First, GCN performs end-to-end learning of feature information and structural information of lncRNAs and drugs, which can comprehensively capture global information and represent node features well. Second, the graph attention mechanism aggregates the neighbor information of nodes according to the attention coefficient to obtain its embedding, which can efficiently represent local neighbor information.

## 4. Discussion

LncRNA plays an important role in carcinogenesis and can lead to the resistance of tumor cells to chemotherapeutic drugs (Wang et al., 2017; Ashrafizaveh et al., 2021), which is an essential factor leading to high cancer mortality (Vasan et al., 2019). Therefore, identifying lncRNA-drug resistance associations becomes crucial for revealing the impact of lncRNAs on the drug resistance of tumor cells. Recently, machine learning has achieved promising results in predicting biomolecular association. However, to the best of our knowledge, little work has been done on machine learning to predict lncRNA-drug resistance associations. In this study, we proposed DeepLDA, a powerful deep learning model based on graph neural network and graph attention mechanism for revealing the potential relationship between lncRNAs and drug resistance. DeepLDA first used known association items to construct similarity networks of lncRNAs and drugs. Subsequently, deep graph neural networks were used for preliminary learning of lncRNA and drug features. Finally, these learned features were input to GAT to learn lncRNA and drug embeddings to predict potential association pairs. Experimental results show that DeepLDA outperforms other machine learning methods in predicting lncRNA-drug resistance pairs on the given datasets. In summary, our proposed a computational model, DeepLDA, that can effectively complete the prediction task of the association between lncRNAs and drug resistance. DeepLDA can provide valuable insights for drug design and open new avenues for lncRNA-related research.

On the one hand, the association between miRNA/circRNA and drug resistance in cancer cells has been confirmed (Leonetti et al., 2019; Xu et al., 2020; Pan et al., 2021; Wang et al., 2022). DeepLDA provides a reference for the study of the relationship between miRNA/circRNA and drug resistance, and the prediction process is as follows. Firstly, GCN learns the features of miRNAs/circRNAs and drugs, which can comprehensively capture global information and represent node features well. Subsequently, the graph attention mechanism aggregates the neighbor features of nodes according to the attention coefficient to obtain their embeddings. Finally, the learned embeddings can be used to effectively predict potential associations between miRNAs/circRNAs and drug resistance. On the other hand, DeepLDA can facilitate the development of targeted therapies. Despite recent discoveries in cancer treatment, the study of resistance to chemotherapy, radiation therapy, targeted therapy, and immunotherapy remains a major challenge. LncRNAs are widely recognized as universal regulators of multiple cancers, such as proliferation, apoptosis, invasion, metastasis, and genome instability (He et al., 2021; Nandwani et al., 2021). Based on this, lncRNAs can be used as therapeutic adjuvants and components of tumor-agnostic therapeutic strategies to improve anticancer responses to existing treatment modalities. Therefore, lncRNA-based targeted therapy can be developed on the basis of DeepLDA to intervene in lncRNA drug resistance.

Despite DeepLDA has significant advantages in predicting potential lncRNA-drug resistance associations, its limitations should be informed. The known association matrix remains sparse. In future work, we will collect more lncRNA-drug resistance associations and employ other feature learning methods to better explore the feature information of nodes to improve the model performance. In addition, we will combine other biological characteristics to carry out lncRNA-drug resistance association prediction analysis to effectively mine the regulatory mechanism of lncRNA.

## Data availability statement

The original contributions presented in the study are included in the article/supplementary material, further inquiries can be directed to the corresponding author.

## Author contributions

MG designed and implemented the method. MG and XS wrote this manuscript. Both authors contributed to the article and approved the submitted version.
